# MUFAs

**DOI:** 10.1016/j.advnut.2026.100725

**Published:** 2026-08-20

**Authors:** Xiaojing Li, Chaodong Wu

**Affiliations:** Department of Nutrition, Texas A&M University, College Station, TX, United States

**Keywords:** monounsaturated fatty acids (MUFAs), nutrients, metabolism, cardiovascular disease, dyslipidemia, diet composition

MUFAs are dietary fats characterized by a single unsaturated carbon bond. Commonly found in olive oil, avocados, and nuts, MUFAs play a vital role in human health. In a previous nutrition note, Mashek and Wu [1] summarized the essential aspects of MUFAs, highlighting their food sources, health benefits, and potential toxic effects, specifically regarding liver outcomes. Ten years later, this update summarizes the impact of MUFAs on cardiovascular outcomes and their application within the broader dietary landscape. Specifically, it emphasizes the importance of food sources, noting the distinct health implications of plant-based compared with animal-based MUFAs, and the influence of overall diet composition.

## Deficiencies

There is no recognized clinical deficiency syndrome for MUFAs in humans. However, an inadequate dietary intake of MUFAs is associated with suboptimal health outcomes such as cardiovascular risks and poorer metabolic health.

## Diet Recommendations

Although there are no established Dietary Reference Intakes for MUFAs, it is recommended to substitute saturated fats and trans fats with MUFAs (and PUFAs) to optimize health.

## Food Sources

Olive oil, avocados, nuts (almonds, cashews, and pistachios), and seeds are the preferred sources of MUFAs. These foods provide additional benefits through antioxidants and fiber.

## Clinical Uses

MUFAs are primarily prescribed through targeted medical nutrition therapy and specialized clinical feeding systems. For metabolic and cardiovascular management, swapping saturated fats for MUFAs is used to help improve insulin sensitivity, reduce systemic inflammation, and lower LDL cholesterol while maintaining or increasing HDL. For critical care and parenteral nutrition, lipid emulsions (which are rich in MUFAs) are used to provide a dense, safe caloric source that is less susceptible to oxidative stress and lipid peroxidation compared with PUFA alternatives, helping to protect immune function during critical illness.

## Toxicity

There is no established tolerable upper intake level, and there are no reports of toxicity in humans.

## Recent Research

Abundant evidence shows that replacing saturated fats (SFAs) or refined carbohydrates with MUFAs, particularly from plant sources, significantly improves lipid profiles and reduces cardiovascular disease (CVD) risk [2,3]. These cardioprotective benefits, often observed in the Mediterranean diet, are driven by lower LDL cholesterol, maintained HDL levels, and enhanced insulin sensitivity. Recent research also suggests that plasma lipid metabolites, such as glycerolipids and sphingolipids, may mediate these effects. However, the source of MUFAs is critical; although plant-based MUFAs are beneficial, the health outcomes of MUFA-containing foods are also influenced by other dietary components.

### Plant-based compared with animal-based MUFAs

Substantial evidence suggests that MUFAs from plant sources (olive oil and nuts) are associated with lower coronary artery disease risk, whereas MUFAs from animal products may not offer the same protection. For example, a 2019 analysis of 2 large prospective cohorts found that higher intake of MUFA-Ps was associated with a 16% lower risk of all-cause mortality, whereas higher consumption of MUFA-As (from red meat, dairy, and eggs) was associated with a 21% higher risk of death [4]. Therefore, both the macronutrient substitution and the specific dietary source are critical determinants in evaluating the clinical efficacy of MUFA-rich interventions.

### Diet composition

Although MUFAs are a primary driver of heart health, the benefits of plant-based foods are derived from a complex synergy of several “nonfatty acid” components. These constituents often explain why plant-derived MUFAs outperform animal-based sources in clinical outcomes. The key components responsible for these health benefits include dietary fiber, phytochemicals, vitamins, essential minerals, and plant proteins. These components have been repeatedly shown to reduce inflammation and improve metabolic outcomes. Whether MUFAs interact with these components individually or collectively remains to be fully elucidated.

### Divergent findings

Despite general consensus, some studies show contradictory results. A Mendelian randomization analysis found no direct association between serum MUFA concentrations and risk of stroke or myocardial infarction. Additionally, some studies have linked plasma MUFAs with a higher risk of coronary artery disease, whereas *n*–3 PUFAs showed a significantly lower risk [5]. In terms of dietary intervention, consumption of high-MUFA diets for 1 y brought about beneficial effects comparable with consumption of conventional high-carbohydrate diets [6]. These discrepancies highlight the need for further research to elucidate the links between MUFAs and CVD risk.

In summary, current evidence supports the health benefits of high-MUFA diets only when emphasizing plant-based sources. MUFAs from animal products do not appear to offer the same cardiovascular protection and may be linked to adverse health outcomes.

## Author contributions

The authors’ responsibilities were as follows – XL, CW: wrote the manuscript; CW: developed the concept.

## Funding

This work was supported in whole or in part by a grant from the National Institutes of Health (DK124854 to CW).

## Conflict of interest

The authors report no conflicts of interest.
